# On Goître and Cretinism

**Published:** 1815-06

**Authors:** Joseph Adams


### Medical and Physical Journal.

6 OF VOL. XXXIII.] JUNE, 1815. [NO. 196.

"For many fortunate discoveries in medicine, and for the detection of nume-
"rous errors, the world is indebted to the rapid circulation of Monthly
"Journals; and there never existed any work to which the faculty in
"EUROPE and AMERICA were under deeper obligations than to the
"Medical and Physical Journal of London, now forming a long, but an
"invaluable series."—RUSH.

*For the Medical and Physical Journal.*

### *On Goître and Cretinism;* by JOSEPH ADAMS, M.D.

IN the few lines with which you indulged me in the last Number of your valuable pages, it was slightly mentioned that the theory of the French physiologist concerning the origin of Goître and Cretinism, is not more remote from the generally-received opinion, than from the one which I have ventured to offer. I shall now endeavour to show that his theory is unsupported by any pathological laws hitherto discovered, or by any well-ascertained observations on the peculiarities of the human race; and lastly, that all the facts he has collected, serve to satisfy me, and I hope your readers, that "Goître and Cretinism are endemial in certain places, from no other causes than an hereditary property commencing in certain individuals, and perpetuated by sequestration and consequent intermarriages."

In answer to the generally-received opinion, that the disease arises from air, water, altitude, or any other known locality, sufficient has already been produced by the American physiologist (Dr. Barton) and the French philosopher. Both have proved such a want of uniformity in effect, as is absolutely inconsistent with the supposed causes. Discovered, as these people are described, in hordes in the Alps and Pyrenees, dwelling in the neighbourhood of, but forming no intimate connection with, families totally free from such peculiarities, how can we ascribe them to local causes? The first error, concerning water, has descended to us from Pliny, and even he speaks of it with an air of scepticism, rarely indulged by that credulous philosopher.* Dr. Barton,

* See lib. xi. cap. 37. Guttur homini tantum et suibus intumescit aquarum quæ potantur *plerumque* vitio.

indeed, imputes the disease to a particular constitution of the air; but, in the note copied from the Treatise on Hereditary Diseases, it has been shewn that there is the same want of uniformity between this supposed cause, and the effect, as in water. The coincidence in the hills of Derbyshire and the Alps and Pyrenees, has strengthed an opinion in favour of an elevated situation as a sufficient cause; but we have seen, by M. Ramond's account, that the disease is not confined to such spots, and Professor Barton found the swelled throat so much more frequent in vallies, or flat countries, that he imputes it to the same cause as induces intermittent fevers.

Let us now consider the force of M. Ramond's argument that this change in the human constitution has been induced by slavery and oppression. There cannot be a doubt of the justice of Homer's remark, that, when man becomes a slave, he loses part of those advantages which should distinguish him from brutes. His external form may be deteriorated by premature and constant labour; his mental faculties hebetated for want of cultivation or exercise; and his moral character depraved, by the necessity of opposing falsehood or cunning to severity and oppression. But will these causes induce a swelled throat, or even idiotcy? Have such changes ever been remarked, from the earliest accounts of slavery in the Children of Israel, to the most modern periods, when the condition of slaves has been traced with so much attention, and with no wish to under-rate its consequences.

It now becomes me to shew how such an effect is to be produced by any hereditary causes. By this I would not be understood to mean that perpetual intermarriages in the same family has in itself any power of deteriorating the race. The contrary is proved by the most authentic records, as well as living examples. That the Hebrews are of one family, cannot be questioned. Throughout all the diversity of condition, and of local residences, which their dispersion has induced, the same countenance is to be traced, with no other variety than in complexion and degrees of symmetry. This nation is the only one that can be traced to such an extent in Europe, or probably in the world, with such family distinctions, and with historical records, under such extraordinary dispersion, because they are, perhaps, the only individuals in Europe who have not gradually amalgamated with the polished nations among which they have emigrated. Family distinctions are, however, sufficiently observable in many states, the members of which have remained stationary, and much secluded from the period of their patriarchal government.

ment. These distinctions must gradually lessen, as the intercourse of the different nations increases; but it will be long before they are entirely obliterated. In the instance of the Hebrews, it must be admitted, that the only effect has been the preservation of a certain peculiarity of features or form, without the production of disease. But goître is a disease; and cretinism, if not an entire, is a partial, privation. We must, therefore, seek for some additional cause to account for the general prevalence of such effects; and I trust it will not be difficult to show that the prevalence of certain diseases, in certain districts, has been imputed to air, water, and situation, which, on a more accurate examination, can only be accounted for by an hereditary cause, little interrupted by external alliances.

Whoever peruses Hippocrates' book, *De aere locis et aquis*, will be convinced that this great philosopher, partly in his travels, and partly from reports, discovered several endemic peculiarities among certain nations, the prevalence of which he imputed to air, water, and locality; but which can only be accounted for by the presumption that the inhabitants, having sprung from one common stock, have, for the most part, retained the same family peculiarity of constitution. To what state of the air can we impute the frequency of hydrocele, even in young children, or a disposition to hæmorrhage or epilepsy, which last, with some other diseases, mentioned in the same section, are known to be hereditary. Yet the prevalence of these diseases, and even the improvement of the human race in beauty and mental faculties, are by Hippocrates imputed to local causes. Not contented with this, he ascribes to the aspect of a town that melody of voice so universally considered as a family or hereditary endowment. These opinions he extends to the inferior animals, imputing the want of horns in the Scythian cattle to the state of the air, though it is now well known that the race called the poll cattle may be propagated in any climate.*

It will, perhaps, be urged, if that we admit goître and cretinism to be hereditary, this can only account for the multiplication of such objects, and not for their origin. In answer to this, I need only repeat what has been demonstrated in the foregoing Treatise, viz. that the origin of hereditary diseases must for ever remain beyond our research; because the peculiarity of constitution must always have commenced in individuals whose parents and remotest ancestors were free from it. In the same place, the provisions were pointed out which seemed made to prevent the universal prevalence of such diseases, and to induce their gradual extinction in families in which they are hereditary. This naturally led to a consideration of those causes by which such diseases are likely to become more generally prevalent. It was shewn that among the inferior animals, peculiarities or varieties, as they are called, occur, which do not exist in either parent, but which may be perpetuated in their offspring. To accomplish this, it is found necessary to seclude the race; and, if we wish to preserve the variety in its greatest perfection, we also withdraw from the flock such as are the least marked. To apply this to the human race, we must refer to that remote period when they subsisted chiefly by hunting or pasturage, and required a larger tract of land for their support. In such a state of mankind, single families, governed by patriarchs, would, from necessity or choice, migrate as often as the district became too populous. On all these occasions the weakest must submit not only to migration, but gradually to seek security in the most secluded or inaccessible places; and, as the general population increased, they would at last, as M. Ramond remarks of the cagots, by the same events, be confined to the most remote and desert spots.

Thus secluded from the rest of mankind, the family must constantly intermarry, and any peculiarity of form or hereditary disposition, would, with greater probability, be perpetuated. If this peculiarity were favourable, it would be preserved with more certainty, because the inclination of each sex would induce alliances with the best favoured, and the most vigorous would live to the age of forming such alliances. Even should the peculiarities be less favourable, they might gradually be lessened by the first-mentioned causes; and the premature death of those who partake most of the hereditary diseased disposition, would be very much increased, if, as was shewn in scrofula, the climate should be such as to induce an early development of the disease in an exasperated form. The operation of the first-mentioned cause would be slow, because we well know that an hereditary peculiarity will apparently cease for a generation or two, and afterwards shew itself in the offspring of a couple entirely free from it. To extinguish it, as far as possible, no means occur but that general intercourse with other communities, which takes place in proportion as the members of each approach the other, or in proportion as migration to and from them is facilitated. Even then, individuals inferior in talents, enterprize, or successful industry, will remain on the spots, and this class will be the last to show any considerable improvement.

Let

Let us then suppose that a family in whom the swelled throat was hereditary, had found it necessary to emigrate. That, from their imbecility, they had sought a secluded spot, and had remained for many generations separated from the rest of the world. In such a community, no cause would exist to lessen the family peculiarity. The disease is not influenced by climate in such a degree as to destroy prematurely those who are most disposed to it. The constant sight would lessen that repugnance to a deformity which would be disgusting to strangers; add to this, at the most interesting period of life, the progress of the disease is often such as would be little noticed where its most advanced stage is perpetually obtruding itself. By such a family, I conceive, the less accessible parts of the Alps and Pyrenees were peopled;—at what period, it is impossible that we should now ascertain. That they were once comparatively few in number, or confined to the most secluded parts of the Alps, we may conclude, as they are unnoticed by any writer of antiquity excepting Juvenal, and that they were frequent in his days cannot be questioned, whatever allowance it may be right to make for his strong propensity to caricature.—Quis tumidum guttur miratur in Alpibus?

This testimony is enough to shew that their origin is much more remote than any period assigned by M. Ramond. Since the perusal of the extract contained in page 209 of your present volume, I have taken some pains to learn all that can be collected concerning the people whom he designates as Cagots, Cacots, or Capots. The French Cyclopædia contains an interesting historical account very similar to M. Ramond's. The writer of the article refers to Marca's History of Bearne for his information, and there is no reason to question the authenticity of the relation. But it is far from clear that the Cagots were either Cretins or Goîtres; or that the Goîtres and Cretins of the Pyrennees and the Alps were at all connected with the race called by the opprobrious epithet which M. Ramond heard pronounced with so much reluctance. It is well known that a term of reproach soon becomes general, when its origin or etymology is often lost. It is not less certain that Cagot is at this time a common term of opprobrium for a hypocrite or bigot, though the French have the same words as ourselves to express such characters. It is, therefore, at least suspicious that M. Ramond, having heard these unfortunate people called Cagots, made it his business to inquire what was meant by the term, and that he has associated the history of a race said to be the remains of sectarian invaders, with another noticed before Christianity was any where established blished. It must, however, be admitted, that, though M. Ramond is probably mistaken, yet much more is necessary to be known before the whole intricacy of the question can be completely unravelled. All, therefore, that can be expected in the present state of France and of Europe, is to make the most of such materials as the best documents afford.

If we admit the causes I have assigned, they explain no more than the probable origin of Goîtres. I know not that the swelled throat is any where associated with idiotcy, excepting in the Alps. It certainly is not in Derbyshire; and the accounts of the American pathologist would induce us to suppose that no such union exists in the western hemisphere. M. Ramond speaks of Cretins as frequent in the Pyrenees. If this be the case, it has not generally been remarked by former travellers. However, as it is probable that Cretinism was not always associated with the swelled throat in the Alps, the same cause, at different times, may have produced the same effects in each. That idiotcy was not general in the Alps in Juvenal's time, may, I think, be fairly inferred, from his not mentioning it, when his whole object was to shew, that in certain districts certain peculiarities in the human race were so common as to excite no particular attention.

Quis tumidum guttur miratur in Alpibus? aut quis
In Meroe crasso majorem infante mamillam?
Cærula quis stupuit Germani lumina.——&c. Sat. xiii. 162.

It is scarcely probable that a single family should have been visited by two such calamities; but it is easy to conceive that the union of two such families sequestered from the rest of the world, may have produced such a race as is described in the Alps. Goître, we are told, even in that part of the world, is not necessarily associated with idiotcy, nor idiotcy with Goître. If conjecture may be admitted, where we have no documents to assist us, it may not be difficult to discover causes for such a mixed race as is described in the Alps.

It has been very well observed by Professor Spurzheim, that the generality of those solitary human beings considered as in a state of nature, have been idiots who wandered from their parents, and have just had understanding enough to procure their own food in the deep recesses of uninhabited forests or rocks. That this may occur on the continent, is easily understood, on account of the immense forests which are preserved for fuel and other purposes, even in the best cultivated countries. It is also certain that there is a propensity in idiots to wander, either from stupid curiosity, or from ignorance of the consequences, or even from an incapacity to recover their right road when among those who use what may be considered their native language. Of this I shall relate two remarkable instances in one family. A couple, one of whom had a mind somewhat above the common level, and the other was equal to the customary business of life, had two children approaching to idiotcy, and another, who, till about the age of puberty, when she rapidly improved, was greatly inferior to those of her age. The condition of the eldest was so evident, that she was removed to the care of a cottager, in a distant part of the country, on a pension which rendered the preservation of her life and her comfortable existence an object of interest to those who had the charge of her. The other, during the period of her imbecility, left her parent's house on a trifling errand into the next street, and wandered from the neighbourhood of Covent Garden to Stepney, before she discovered the necessity of or had courage to inquire her way. Both these had the free use of their native language. The third, a boy, at the age of about ten, left his parent's house at a moment when he was not attended to, and wandered into the fields at a considerable distance from home. In this condition, he sat down, till he was discovered by a hair-dresser, who, struck with his tender age, a countenance of distress, and the neatness of his apparel, endeavoured to learn his history. The boy was unable to explain himself. His condition, however, excited the compassion of his discoverer, who took him to his house. Throughout the whole evening and the following morning, the unfortunate foundling was observed to utter only one intelligible sound. The hair-dresser, in the morning, amused all his customers with the adventure. One of them advised him to apply to every shop he saw answering to the term which escaped from the boy; and to inquire at each concerning the probability of any family to whom he might belong. In this manner the parents were, after two or three days, discovered, and the boy restored. From that time he was never able to give an account of any event that passed during his absence; and the only recollection he ever shewed was, on the following evening, bursting into a flood of tears.

When idiotcy occurs in an English family, the consequence depends much on the birth of the object. If of the higher rank, almost the only solicitude is to prevent the disgrace of the family by an improper marriage, and this, if the imbecility is very considerable, is not difficult. In inferior rank, a male idiot is made useful as far as his abilities will will permit; but he never shews any partiality to the other sex beyond a sense of gratitude for those attentions which, to their honour, they bestow from motives of unmixed compassion. Females of this description rarely quit their mothers, unless consigned to the work-house. The insular form of our country, and its generally-cultivated state, prevents the occurrence of those wild human beings, as they are called, of which we often hear on the continent.

Should a wanderer of this description find his way to a proscribed nation, which is conscious of a peculiarity sufficient to seclude them from the rest of the world, we cannot doubt that a feeling, if not of respect, at least of sympathy, would be excited in favour of one to all appearance of a superior order, perfectly inoffensive, and whose want of intellect would for some time be confounded with his ignorance of their language. When we recollect the history of the family related by Haller,* and also that there is nothing in climate to shorten the lives of these unfortunates, the consequence may easily be conceived if such an event should occur even once in a succession of ages.

From these considerations, I should conclude that the whole race of Goîtres are the product of a single family, and that idiotcy has probably been since introduced among them by the causes above assigned. That, from the general disgust they excited, they have been driven to rocks at one time almost inaccessible. That idiotcy has been since introduced among them, either by the event above mentioned, or that, having occurred in the family, it has been perpetuated from inattention, or from that sympathy which common distress so naturally excites. If this is not the true history of the origin and present condition of that unfortunate race, it appears at least a sufficient cause, and may, perhaps, be found the only conjecture consistent with sound physiology, applied to all the facts on which we can depend. Aware, however, how much more easy it is to object to the opinions of others, than to establish one's own, I trust what has been offered will only be considered as conjecture, requiring for confirmation a much more comprehensive knowledge of the subject. For want of such knowledge, I shall conclude with the judicious motto selected by Dr. Barton, which may serve as an apology for us all:—

"I know but one class of people who never err, those who do nothing, observe nothing, and make no experiments."—Fontana.

* See Treatise, p. 88.

The

The following extracts, with the few remarks on each, could not easily be introduced in the paper without interrupting the chain of reasoning. In their present form, they afford an opportunity of showing the authorities alluded to, and of further illustrating the doctrine, after the reader is made acquainted with the general conclusion.

For an account of the Cagots, I am indebted to the French Encyclopædia in the London Institution.

" Cagots ou Capots c'est ainsi dit Marca dans son histoire de Béarn qu'on appelle en cette province, et dans quelques endroits de la Gascoyne, des familles qu'on pretend descendues des Visigoths qui resterent dans ces cantons apres leur déroute generale. Ce que nous en allons raconter est un exemple frappant de la force et de la durée des haines populaires. Ils sont censés ladres et infects; et il leur est defendu par la coutume de Béarn sous les peines les plus séveres, de se mêler avec le reste des habitans. Ils ont une porte particuliere pour entrer dans les Eglises et les siéges separés. Leur maisons sont ecartées des villes et des villages. Il y a des endroits ou ils ne sont pas admis a la confession. Ils sont charpentiers et ne peuvent s'armer que des instrumens de leur metier. Ils ne sont point recûs en temoignage on leur faisoit enciennement le grace de compter sept d'entré eux pour un temoîn ordinaire. On fait venir leur nom de *caas Goths*, chiens des Goths. Cette denomination injurieuse leur est restée, avec le soupçon de ladrerie, en haine d'Arianism dont les Goths faisoient profession. Ils ont été appellés *chiens* et reputés *ladres* parce que ils avoient eu des Ancêstres Ariens. On dit que c'est par un chatiment semblable a celui que les Israelites enfligerent aux Gabaonites, qu'ils sont tous occupés en travail des bois. En 1460 les etats de Béarn demanderent a Gaston d'Orleans Prince de Navarre qu'ils leur fut defendu de marcher pies nuds dans les rues sous peine de les avoir percés et enjoint de portér le pie d'oie ou de canard sur leur habit. On craignoit qu'ils n'affectassent et l'on pretendoit annoncer par le pie d'un animal que se lave sans cesse, qu'ils etoient immondes. On les a aussi appellés *Geziatins,* de Giezi, serviteur d'Elisée, que fut frappé de lepre. Le mot Cagot est devenu synonyme a *hypocrite.*"—*Encyclopédie*, *Paris,* 1751. Article *Cagots.*

Ramond, whilst the general import of his argument would lead us to suppose that Goitre and Cretinism are confined to a particular race, or, in other words, that they are hereditary, seems much at a loss to comprehend how a nation of warriors should be so degenerated. This he attempts to account for by supposing that oppression had produced such changes. Besides the arguments above offered against such an opinion, we may see, in Ambrose Paré, to whose writings Ramond refers us, that the leprosy of which the Cagots were suspected had not the least connection with goître. The two diseases are spoken of in distant parts of his works.*

* In book 2, chap. ix. under the term Ladrérie, Ambrose Paré speaks

M. Dralet, after objecting to the theories of all his predecessors, imputes the disease to the great difference between the temperature of the day and night in certain mountainous parts; yet he is forced to admit that many of the inhabitants escape. This he explains by their better condition of life, observing, that most of the goîtres are wood-cutters or *charpentiers*. It is surely much more reasonable to suppose that these occupations should be confined to certain families, than that such necessary employments, without slavery, should be attended with abject poverty, or even that abject poverty should induce such effects.

M. Dralet, in the character of *Conservateur des Eaux et Forêts de la 13 Division*, visited the Pyrenees two years after Ramond, and probably had larger opportunities of examining the character and habits of the nation. By comparing the various parts of his work, one might almost suppose that he had some confused notion that the Goîtres were once a colony of refugees from the Cagots; and that the Cagots, instead of being the descendants of the Goths, were rather the aborigines of the country.

"Pour procéder avec ordre, mettons en première ligne les Basques Français, que l'on divise en Labourdins, Bas-Navarrois et Souletins. Leurs ancêtres, descendans des Ibériens, arrêtèrent les Romains dans le cours de leurs conquêtes, résistèrent aux Suèves et aux Visigoths, opposèrent des armes victorieuses à celles de Clovis et de ses enfans, et rendirent vaines toutes les attaques que dirigèrent contre eux les Sarrasins. Généreux autant que braves, ils ne se bornèrent pas à défendre leur pays et à en étendre les limites, ils servirent Viriathus, les Numantins, Sertorius, et Pompée, et furent toujours favorables à quiconque se présenta comme victime de l'oppression, ou armé pour la cause de la liberté.

"C'est l'amour de cette liberté qui attacha constamment les Basques au séjour des montagnes, où la nature offre tant de moyens de défense et tant de facilités pour rendre vaines les poursuites d'un ennemi puissant. Ce peuple, pasteur et guerrier, évita avec soin tout ce qui pouvait apporter quelque changement dans sa manière d'être. A l'attention de ne point se mêler avec d'autres peuples † il joignit celle de ne permettre aucune innovation dans son langage, dans son culte et dans ses coutumes. C'est ainsi qu'il a conservé, d'âge en âge, la pureté de son sang et la simpli-

---

speaks of the Cagots in Britany as exhibiting no external character of leprosy, and as a well-favoured people. Did the influence of the priesthood oblige him to call them lepers?—Goîtres are mentioned with reference to the bronchocele of the ancients, in livr. vii. cap. 9, without any reference to the Alps or to Cretinism. Can any of your readers inform us who was the first writer that speaks of Cretinism among the Goîtres? A. Paré wrote about the middle of the 16th century.

"† De temps immémorial il existe dans les pays basques des familles cité de ses mœurs."—*Description des Pyrenees, par M. Dralét,* vol. i. p. 163.

If the reader compares the text and notes of the above extract, in the one he will find the Basques described in terms almost encomiastic; in the other the same people, or a race similar in their physical and moral qualities, are called Cagots, and said to be with difficulty admitted into society. Mutual antipathies may have existed for a succession of ages, till the causes have been forgotten, and nothing remains excepting a tradition by which the families are distinguished. But such can never be the case, where the figure and want of intellect are so manifest as in goîtres and cretins. In the chapter from which the above extract is taken, no mention is made of this unfortunate people; but in the succeeding chapter they are described very much at large, and with epithets which M. Ramond makes synonymous with cagots. Without attempting to unravel these difficulties, I shall content myself with transcribing the following passage.

"Un tel état de misère rendit toujours les goîtreux à charge à la société; on évita dans tous les temps de s'allier avec eux. Un sang vicié fut transmis de père en fils, et ne put se mélanger qu'avec un sang aussi vicié. Ainsi se formèrent ces races de crétins, que l'on appelle aussi dans les Alpes et les Pyrénées *caffos*, en Auvergne *marons*, et c'est, n'en doutons pas, à des circonstances à peu près semblables que les *cacous* ou *caqueux* de la Bretagne et les *colibets* de l'Aunis et de la Rochelle doivent leur origine. Ces circonstances donnèrent naissance aux maladies des premiers habitans, qui furent fixées par la misère dans certaines familles dont on voit encore les rejetons.

"Il n'est aucune précaution que l'on n'ait prise anciennement pour empêcher que ce mal n'étendit ses ravages. Les crétins

---

milles étrangères, dont les individus sont connus, les uns sous le nom de *Cascarots*, les autres sous celui d'*Agots*.

"Les Cascarots ou Bohémiens [gypsies] sont réputés vagabonds, et ont été mis de tout temps sous une surveillance particulière de la police.

"Les *Agots* ou *Cagots* sont domiciliés; ils ne diffèrent des Basques d'ancienne origine ni sous le rapport du physique, ni sous celui des mœurs. On ne les connaît que par la tradition, qui indique que telle ou telle famille est *agote*, et que tel ou tel individu lui appartient. Les Agots passent pour être descendans des Goths. Ils vivaient autrefois dans un tel état d'abjection, qu'on ne leur permettait point de passer le péristile des églises. Quoique le temps ait beaucoup adouci le préjugé qui les poursuivait, il est encore très rare, dans les campagnes, que l'on contracte des alliances avec eux. Il n'en est plus de même dans les villes; les Agots sont confondus avec les autres citoyens, et l'on ne s'occupe plus d'eux."

 avaient avaient une place séparée dans les églises; ils y entraient par une porte particulière, et avaient un bénitier uniquement destiné à leurs usages; de telles mesures se multiplièrent au point qu'en 1460 les états de Béarn voulaient qu'il fût défendu aux crétins de marcher pieds nus dans les rues."—*Dralet*, vol. i. p. 188.

I have ventured to suggest that there is probably no other connection between goître and cretinism than the accidental circumstance of an union between two such families. Dr. Reeve, in an ingenious paper published in the Philosophical Transactions, after describing several cases of cretinism, unaccompanied with goître, assures us that

"There is no necessary connection between goître and cretinism, notwithstanding the assertions and ingenious reasoning adduced by Foderé. That it is probable the one has been assumed as the cause of the other, from the enlargement of the thyroid gland being a frequent occurrence in cretins; and as it forcibly strikes the observer from the deformity it occasions, this strong impression may have converted an accidental, though frequent, occurrence, into a general and necessary cause. Cretinism is frequently observed without any affection of the thyroid gland, and that gland is often very much enlarged without any affection of the intellectual faculties. There seems [continues he] to be some similarity between cretinism and rickets, as they both take place in infancy, are both characterised by feebleness of body, and sooner or later by feebleness of mind, and they both affect males and females equally; but there is no sort of connection between persons afflicted with bronchocele in England, and with rickets. For, although it might be granted, that there is some delicacy of frame in females about the period of pubescence, when bronchocele usually occurs, yet neither irregular formation of the bones, nor weakness of the intellectual powers, are common symptoms attending bronchocele in Britain."

See also Memoirs of the late Dr. Reeve, Edin. Med. Journal, vol. xi. p. 254.

Where, we might ask this ingenious and candid writer, is the similarity between cretinism and rickets; or which are the authors who speak of rickets as attended with *feebleness of mind?* At the close of the paper, Dr. Reeve adds, as a presumptive argument in favour of his opinion, that Glisson describes rickets in Britain about the time that Plater mentions cretinism. But this only shows the improved progress of our art about the same time, in consequence of which, facts before unnoticed, or only noticed by the vulgar, began to attract the attention of philosophers. We are not to suppose that angina pectoris was less common before Dr. Heberden described it, or that mumps and croop began to exist when the improved state of medicine in North Britain brought them into notice, and procured us accurate descriptions of each.

It is remarked both by Dr. Reeve and M. Dralet, that in proportion proportion as the country improves in wealth and cultivation, the diseases are less prevalent. This is extremely probable, because, in proportion as men learn the charms of an improved mode of life, they are cautious not to engage in the expences of a family without a prospect of decently supporting it: at the same time an improved taste arises, and even a view to posterity, all which will tend to check the increase of population, and to improve the succeeding generations.

It is to be regretted that some of our most ingenious writers should fancy themselves obliged to account for the origin of diseases which afterwards prove hereditary. Is it more remarkable that a diseased disposition should be perpetuated than an actual monstrosity? In the less complicated animals, where we have opportunities of making experiments, it has been remarked, that peculiarities, the causes of which we never attempt to explain, may be easily preserved. Besides those which are well known, a single-hoofed boar, as mentioned by Bradley,* is now in the possession of Sir Joseph Banks. From a sow with the cleft hoof, a litter is produced, the individuals of which are formed, some like the sire, and others like the dam. Probably nothing more is necessary to produce a race of single-hoofed swine, than the selection of all those which are thus formed. In my treatise, many instances are produced of similar peculiarities of form in man which have proved hereditary, and that they most commonly occur in parts of little importance.† If no one attempts to account for their first appearance, why should we think it necessary to ascertain the origin of hereditary diseases? The following attempt of Dr. Darwin shows the danger of uniting poetry with pathology.

"In many cases, however, there is no appearance of the disposition to epilepsy or insanity of the parent being transmitted to the progeny. First, where the insanity has arisen from some violent disappointment, and not from intemperance in the use of spirituous liquors. Secondly, where the parent has acquired the insanity or epilepsy by habits of intoxication after the procreation of his children. Which habits I suppose to be the general cause of the disposition to insanity in this country. See Class III. 1. 1. 7.

"As the disposition to gout, dropsy, epilepsy, and insanity, appears to be produced by the intemperate use of spirituous potation, and is in all of them hereditary; it seems probable, that this disposition gradually increases from generation to generation, in those families which continue for many generations to be intemperate in this respect; till at length these diseases are produced; that is, the irritability of the system gradually is decreased by this powerful stimulus, and the sensibility at the same time increased, as explained in Sect. XXXI. 1. and 2. This disposition is communi-

* Philosophical Account of the Works of Nature, page 91.

† See Treatise, pages 67 and 68.

cated cated to the progeny, and becomes still increased, if the same stimulus be continued, and so on by a third and fourth generation; which accounts for the appearance of epilepsy in the children of some families, where it was never known before to have existed, and could not be ascribed to their own intemperance. A parity of reasoning shews, that a few sober generations may gradually in the same manner restore a due degree of irritability to the family, and decrease the excess of sensibility.

"From hence it would appear probable, that scrofula and dropsy are diseases from inirritability; but that in epilepsy and insanity an excess of sensibility is added, and the two faulty temperaments are thus conjoined."—*Zoonomia,* vol. iv. p. 63, 3d edit.

The most unexceptionable part of the above extract is, that the author admits a *parity of reasoning* between the different parts of his theory: that is, that no part is ascertained. Whilst we admit that the whole is much more poetical than philosophical, we cannot help regretting that a writer abounding with so much practical information, should attempt more than human faculties can ever accomplish.

M. Fodéré imputes the goître to a warm and moist atmosphere, having found it on the south-western side of the Alps. Ramond and Dralet assert that it is on the northern aspects of the Pyrenees where the atmosphere is colder. Fodéré shows as little arrangement in his mode of reasoning on this subject as most other men who attempt to account for, instead of exploring, the phenomena of nature. After ascribing the disease to the above local causes, he attempts to explain the manner in which it becomes hereditary. The following are his words:

"'Si le goître n'est qu'accidental et qu'il n'y ait qu'un des parens affecté, les enfans ne naissent pas goîtreux.

"'Ils naissent, au contraire, goîtreux, si, de père en fils, un goîtreux a épousé une goîtreuse pendant deux générations, et dans un pays où le goître est endémique: la troisième géneration, l'enfant qui naît est non seulement goîtreux, mais il est encore crétin.

"'Un pére malsain, rachitique, à demi-crétin, marié à une goîtreuse, produit des enfans goîtreux à la première génération.' *Foderé,* pag. 69."—*Dralet's Description of the Pyrenees.*

In a place where, according to M. Foderé, the causes are constantly operating, how should we determine whether the disease is casual or hereditary? If we may believe that his remarks have any foundation in actual experience, it would be the duty of every woman free from the disease to marry a goître, in order to secure her offspring from the local effects of the south-west wind. Next we are told that the union of goîtrous couples for a certain number of generations produces cretins, and this with much more certainty than an English jockey would calculate on the properties of a horse, notwithstanding notwithstanding the advantages he possesses, by the early maturity of that animal, and the accuracy with which his pedigree may be traced. I shall conclude with remarking, that few writers who impute goître to local causes, entirely escape admitting that it is also hereditary. If two such causes really existed, their constant co-operation in producing a disease which neither shortens life nor renders the subject less prolific, would not only account for its prevalence, but must, by degrees, render it universal in the districts. But the disease is said to be on the decline. This is easily understood of an hereditary disease, as has been before explained; but it is inexplicable if that hereditary disease originates in local causes. I say nothing of poverty and filth, because they do not appear necessary to account for the phenomenon, and because such effects are never produced by these causes in other parts of the world.

*Hatton Garden.* JOSEPH ADAMS.

---

*For the Medical and Physical Journal.*

